# Book Reviews

**Published:** 1804-12-01

**Authors:** 


### CRITICAL ANALYSIS

OF THE

### RECENT PUBLICATIONS

ON THE

### DIFFERENT BRANCHES OF PHYSIC, SURGERY, AND MEDICAL PHILOSOPHY.

---

*A Dissertation on Gout; exhibiting a new View of the Origin, Nature, Cause, Cure and Prevention of that afflicting Disease; illustrated and confirmed by a variety of original and communicated Cases; by* R. KINGLAKE, M.D. &c. &c. 8vo. pp. 348. London, 1804.

THIS Dissertation, in so many distinct sections, treats of the "Origin of gout, the nature and constitution of gout; its remote and proximate cause, the cure of gout, and, lastly, its prevention." Some of the opinions of Dr. K. on this subject have been already laid before our readers, in several communications and replies to opponents in former Numbers of our Journal; which he has here republished in an Appendix. This part, which occupies rather more than half the volume, contains also a considerable number of letters, written by other practitioners, some of which are original communications to Dr. K. and some have been published in the Journal.

Those opinions on which Dr. K. appears to lay most stress, may be collected from the following passages, extracted from his recapitulation,

"The earliest records of medicine attest the existence of gouty inflammation; and it has been uniformly considered, rather as a remedial, than a morbid affection.

"This erroneous opinion of its nature, and healthful influence, has survived the correction which enlightened reason afforded to the small-pox, the plague, and other forms of inflammatory diseases, in which stimulant treatment, and inviting and protracting the distemper on the surface, were also adjudged to be the only safe and efficient means of cure; but which unprejudiced observation ultimately proved to be fatally hurtful, and that the very reverse was the most salutary that could be adopted.

"The nature of gout is purely inflammatory, and possesses no peculiar or specific properties to distinguish it from common inflammation, but what are referable to the structure or organization of the affected parts.

"The seat of the gout is exclusively in the ligamentous and tendinous fabric; the texture of which, when inflamed, affords, all all that is peculiar or characteristic of gout. This fabric therefore is necessary to the constitution of what is called gouty inflammation, which evinces that it cannot occur on any of the visceral or vital organs, as these possess nothing of the ligamentous or tendinous structure.

"The several appellations of gout, rheumatism, and sprain, are only nominally different; they, in fact, describe identity of affection. Any external variation which may present in the degree and progress of the disorder does not alter the fundamental sameness of the disease, which consisting in an inflammatory irritation of the ligamentous and tendinous structure, will exclusively remain such, however variously and capriciously denominated.

"The origin of gout must necessarily be always local, as it can only arise in inflammatory affection of the ligaments and tendons, which are stationed almost exclusively at the joints, and are not co-extended with the system. The peculiar seat of gouty malady at once chains it to the ligamentous and tendinous structure, and gives an undeviating resemblance to its external character. It necessarily originates in every instance in the same natural circumstances, and therefore invariably denotes its existence by the most unequivocal symptoms.

"The diffusion, or propagation, of gout, from the affected joints, is governed by associative or sympathetic influence of motive power; but when arrested on any particular organ, as the brain, stomach, or bowels, it is not characteristic gout in those parts, but simple irritation; the ligamentous and tendinous structure, necessary to gouty inflammation, being wanting in those vital organs. Nor does the local irritation of gout subside by sympathetic distribution, but in the proportion as the severity of pain may have harrassed and exhausted the systematic strength. The consequent general debility will then reduce the local inflammation, and occasion those painfully irregular or agitated motions (usually termed spasm) over the system, which always endanger life, and do often actually terminate in death. Gout, therefore, cannot be strictly repelled; and when it subsides, and the system becomes affected, it is the consequence of extreme reduction of constitutional strength, by protracted pain, and which might have been prevented by its seasonable relief.

"The general health can suffer no other injury from gouty inflammation, than what is occasioned by the debilitating and distempering influence of durable irritation; its early removal therefore renders the disease both mild and safe.

"The general symptoms of gouty inflammation will depend on the temperamental, habitual, or morbid excitability of the system at large, or of any particular organ. If equal health should pervade the frame, no serious impression need be dreaded; but if distempered excitability should have fixed its abode on any particular part, the accumulated weight, or concentered force of sympathetic irritation, may so heavily befall it, as to manifest every every degree of violence from transient to inflammatory excitement. But whatever be the situation or extent of the morbid impression, its nature will be conformable to the structure and office of the affected part, and not to the character of gouty, or ligamentous and tendinous inflammation.

"The protracted or natural duration of gout tends to produce irreparable local derangement on the part affected, and a state of the system promptly susceptible of common causes of morbid impression. The diseased joints will, by its long endurance, become contracted, and be farther distempered by altered structure, by calcareous or osseous, and other vitiated secretions; while the general motive powers of the system will be tremblingly alive to every occurring irritation. This deep and complicated mischief is only to be obviated by an early and radical removal of the disease.

"Gouty inflammation, like most other diseases, acquires by frequent recurrence a facility of return, which soon becomes habitual. The ordinances of health owe much of their fixation and uniformity to the influence of incessant or uninterrupted continuance; those of disease become similarly radicated by long usage. It is therefore of vast importance in a curative view, that an ailment should not become habitually inveterate, and that the gout should, in every stage of its duration, be circumvented and subdued in the most expeditious and efficacious manner, to obviate its familiar or customary establishment.

"Excessive heat has been alleged to be the proximate cause of gout, or to be the disease itself; which obviously suggests as an appropriate indication of cure, an early and unremitted endeavour to allay the morbid temperature on the affected parts, and that the means of reducing it should be proportioned or commensurate to the violence of the disease.

"Cold water is the universal boon of nature, the common medium of salutary temperature, as derived from the repulsive or motive conditions of the atmosphere; its uniform application, therefore, to parts suffering under gouty inflammation, by either ablution, showering, or immersion, is a remedy as efficacious as simple for that malady. Inveterate cases, or the high temperature of tropical climates, may (though very rarely) require the aid of artificial cold, obtained from a solution of neutral salts in water, effectually to extinguish the inflammatory heat. The application of diminished temperature should be uninterruptedly continued, until the painful sense of heat be reduced. This usually happens within forty-eight hours, almost invariably before the expiration of seventy-two hours. The avoidance of all dietetic, medicinal, and mental excitement, at the same time, would likewise greatly co-operate in the intention of cure.

"Costiveness should be removed, or obviated, by occasional laxatives. Neither purging, nor an increase of any other evacuation, tion, is necessary, and by breaking the general strength may prove hurtful.

"The convalescence of gout will require the same attention to further and confirm its progress, as is necessary in that of other violent diseases. A well-conducted plan of nutriment should be regarded as of the first importance in expediting and ensuring perfect recovery. Local friction also, and even topical warm bathing, would be advisable, if an unyielding sense of either torpor or coldness should prevail on the affected part.

"Should a relapse actually occur, it should be treated as the original attack, with such adaptations as the circumstances of aggravated or diminished violence may require.

"Gouty ailment may be either obviated, or arrested in its forming stage, so as to prevent its developement, by resisting the earliest approach of excessive temperature, either generally or locally, by topical cold, aqueous dilution, abstemious diet, cool apartments, avoidance of costiveness, and every other source of morbid excitement, whether universal or partial."

Improvements in medicine are made more slowly than in other sciences, because they are opposed by self interest and prejudice. It becomes necessary therefore, from time to time, to recall the attention of the profession to the errors and prejudices of former practitioners. This appears to have been Dr. K's view in his present work; and there is no doubt that he will have many admirers and many opponents, so that the cause of science cannot fail to be promoted.

---

### *Dr. Rowley's Scholæ Medicinæ,*

Concluded from pp. 469—473 of the last Number.

After the history and chronology of medicine down to the 19th century, Dr. R. addresses a short admonition to students, respecting the best mode of acquiring a correct knowledge of the different branches of the science.

The Second Part, which may be considered as the principal one, and which occupies 104 pages of letter-press, besides the numerous plates, is devoted to the several branches of anatomy. Novelty, or originality, is not to be expected on these subjects; judgment in selecting and arranging, perspicuity in explaining, and a happy illustration by good plates, are all that can be expected; and in these, Dr. R. has not been defective. We discover in every part of his work a great solicitude to consult the memory of the student; and in order to do this, he disposes his subjects in a tabular form, when it can be done conveniently, and indulges in frequent repetitions, for the sake of placing the facts in various points of view, or recapitulating what is most necessary to be remembered. An instance of this kind occurs in the beginning of the Third Part, where we find a Compendium or Abridgment of the Second Part. This is followed by an abridged Physiology, Pathology, and Symtomatology. The work is concluded by an account of the Marylebone Infirmary, considered as a School of Medicine.

---

*A Medical*

*A Medical Glossary, in which the Words in the various Branches of Medicine are deduced from their original Languages, properly accented and explained.* By W. Turton, M. D. 4to. pp. 622. London, 1797.

As the author of this correct and useful work understood his own views and objects in publishing it better than any other person, we prefer his account of them to any we could give.

"Medicine, like all other arts, has its distinct family of terms and idioms, conveying meanings peculiar and appropriate to its several branches; and the very numerous sources from which these have been collected, have made it not easy for its professors sufficiently to understand the language of their science.

"I have therefore brought together such as usage has fixed, or learned men have adopted, and have contented myself with deducing them from their proper roots, determining their pronunciation, and simply defining them.

"The unmeaning jargon of Paracelsus and his followers I have purposely omitted, and have been solicitous to preserve those compound words used by the physicians of the Greek school, most or all of which are scattered about in the writings of succeeding ages. My authorities are chiefly derived from Blanchard, Castellus, Minshew, Schindler, and Golius.

"That such a work is useful, will perhaps be more readily admitted than that it has been usefully executed; but he that has laboured long in attempting to remove the obstructions to science, is not willing to add despondence to his difficulties, and to believe that he has laboured in vain."

The modesty of the title, the correctness and perspicuity of the definitions or explanations of the words, almost disarm the hostility of criticism, if we were ever disposed to indulge in it; but we think no Lexicographer has a right or power to say, this is hereafter to be the language of these branches of science. Dr. Johnson, in his Dictionary, has purposely omitted many words which ought to have been retained, because he thought the English language better without them, or because he did not find such authorities for their use as he approved of. Dr. T. has imitated his example; and this is our only objection to the Glossary.

---

*Pharmacopœia Medici Practici Universalis, sistens Medicamenta Praeparata et Composita, cum eorum Usu et Dosibus. Auctore* F. Swediaur, M. D. Two Vol. pp. 501. London, 1803.

This valuable compendium of practical pharmacy well merits the attention of the medical public from the pains which obviously have been taken in the selection, and the general accuracy with which the precepts for the preparation of the various articles of Materia Medica are laid down. It will also the more interest the English practitioner, as it contains some articles not generally in use, use, and some varieties in the preparation of several of the substances used in medicine.

The Preface begins by asserting the originality of the present compilation of prescriptions, for the greater number of which he acknowledges his obligations to the experience of the successful practice of many of the London Hospitals. Indeed, in this point of view, we apprehend that his work will be still more acceptable to the French practitioners than to our own. The nomenclature of the chemical preparations is that which is now universally adopted by chemists; in the vegetable kingdom the Linnæan is strictly followed. Among other alterations the term *terebinthina* is substituted for *balsamum* (for example, *terebinthina copaiferae officinalis*, instead of *balsamum copaivae)* and with prudent caution the name *tinctura sedativa* is made to supply the place of *T. opii.*

We pass over the author's severe censure on the (supposed) Hippocratic practice, which renders the physician rather a calm spectator of the progress of disease than a vigorous opposer of its ravages, and proceed to a short view of the contents of the work itself.

The purely chemical part of Pharmacy, the preparation of the acids, alkalies, neutral and earthy salts, begins the volume. Among the less common of these articles of Materia Medica are Wertendorf's radical vinegar, made by distilling acetite of soda with sulphuric acid; the citric acid concentrated by frost and mixed with sugar to a dry lemonade; the oxygenated muriatic acid; the phosphoric acid; the sulphite of soda; and the citrates of all the alkalies. Whether many of these will prove permanent acquisitions of value to the Materia Medica appears very doubtful. In a very few instances the directions for the preparation of these salts are defective, but in general they are given with great precision and accuracy. The following quotation will serve as examples of the author's manner of describing, as well as his style of writing that language, the use of which he so strongly insists on in his Preface.

"ACIDUM CITRICUM.

"SYN. *Acidum citri depuratum s. concentratum; acidum limoniorum; succus citri s. limoniorum concentratus.* G. *Acide citrique.*

"℞. Succi fructus citri medicæ maturi expressi et probe defæcati quantum placet.

"Expone in vase vitreo in balneo aquæ gradui caloris 212° th. Fahr. (80° R.) unde pars ejusdem coagulatur, qua abjecta acidum remanens concentratum et purificatum in vasis probe clausis longo tempore bonum conservari potest.

"ACIDUM CITRICUM, gelu concentratum.

"℞. Succi ex fructu citri medicæ maturo in loco ejus natali expressi et probe defæcati quantum placet.

"Expone gradui frigoris inter 23° et 26° th. Fahr. (inter 3 et 4° infra zero th. R.) constanter et diligenter tollendo glaciem prout formatur; vel pertundendo glaciem effunde succum concentratum tratum in aliud vas, et sic repetitis vicibus congelationem repete, ita ut non nisi una tertia pars succi remaneat, qui in vasis probe repletis et clausis longo tempore sanus servari potest.

"Si hujus succi sic concentrati pars una cum partibus sex sacchari albi probe siccati et pulv. misceatur lentis intervallis ita ut saccharum post singulam instillationem succi tempus siccandi habeat, dein ad finem tota massa trituretur, obtinetur *limonada sicca*, quæ in vasis probe clausis usui servetur.

"Pars hujus pulveris in aqua soluta dat limonadam gratam ex tempore quando placet parandam.

"N. B. Prof. Vauquelin, observavit gummi quodcumque in aqua solutum, in acidum citricum mutari, si per hanc solutionem gaz acidum muriaticum oxygenatum transire cogitur.

"ACIDUM MURIATICUM, concentratum.

"SYN. *Acidum muriaticum fumans; spiritus salis fumans Glauberi; acidum salis; spiritus salis marini acidus.* G. *acide muriatique.*

"Gravitas ejus specifica ad pondus aquæ destillatæ sit ut 1,170 ad 1,000.

"℞. Muriatis sodæ siccati et pulv. libras quatuor,

"Inde in retortam tubulatum quæ in balneo arenæ locata jungatur cum apparatu destillatorio *Woulfiano*, in quo prævie aquæ destillatæ unciæ sedecim distribuantur. Commissuris omnibus probe luto obductis, postquam lutum ubique bene siccatum fuerit, adjice per aperturam tubuli lente et caute:

"Acidi sulphurici concentrati, libras duas. Postquam effervescentia subsedit, ignem sensim administra, donec massa bulliat; gaz acidum quod in primum excipulum transit, cum aqua destillato ibidem præsente sese jungens format *Acidum muriaticum concentratum* perfecte limpidum et fumans. Vapores in secundum et tertium excipulum transeuntes, ibique aquæ sese jungentes *Acidum muriaticum dilutum* constituunt quod impregnando majori quantitate vaporum acidorum, æque concentratum reddi potest ac primum.

USUS, aquâ *diluti*: Ischuria renalis; dysuria.—*Concentrati* cum melle *externus*: Aphthæ.—*Item* ad varia præparata pharmaceutica. — Forma vaporum ad putrida aëris effluvia corrigenda."

After these follow the very important class of metallic salts. In the list of metals gold is still retained, very inconsistently in a book which so confidently assumes the merit of excluding all antiquated absurdities; but though the name is retained, the single preparation of this metal is omitted. Under each metal the oxyds are first described, then the salts, and lastly the sulphurets. We shall only notice the rarer of these. M. Vauquelin's convenient method of preparing the *martial ethiops* or *black oxyd of iron* is the following. Mix two parts of fine iron filings with one of the *red oxyd of iron (crocus martis)*, heat them in a covered crucible with a strong fire for an hour, and when cold reduce the coherent mass to a fine powder, which is the black oxyd. The old and well known mode of making the same preparation by moistening iron filings with water and long exposure to air (originally Lemery's) is here introduced with trifling variation, and is ascribed to one Cavezalli, who, it would seem, has had the assurance to claim the invention.

In the important subject of mercurial preparations the author is already favourably known to the public, having formerly, in his Pharmacopœia Syphilitica, exhibited a very full view of the various useful salts and oxyds of this metal. *The black oxyd of mercury by triture, or by ammonia* is not commonly met with in our shops. Its preparation is thus described:

"OXYDUM HYDRARGYRI nigrum.

"SYN. *Æthiops per se; æthiops mercurii simplex; mercurius praecipitatus niger; turpethum nigrum; calx mercurii nigra; mercurius extinctus; mercurius gummosus; mercurius alcalisatus; hydrargyrum cum creta.* Ph. L. *Mercurius solubilis.* HAHNEMAN. *Hydragyrum oxydulatum nigrum.* Ph. B. G. *Oxide mercuriel noirâtre.*

"℞. Hydrargyri purificati quantum placet. In vase ferreo per plures menses, vel tamdiu continuo agita, aëre sæpe renovato, donec in pulverem griseo-nigrum mutetur.

"*Vel.* ℞. Nitratis hydrargyri liquidi libram unam. Aquæ destillatæ libras decem. Instilla paulatim, Ammoniacæ quantum satis; Id est tamdiu nec ultra, quam præcipitatum inde enatum colore nigro apparet. Pulverem nigrum præcipitatum aquæ destillata probe ablutum sicca, et in vase vitreo clauso in loco obscuro usui serva.

"*Vel melius,* Methodo SCHULTZE.

"℞. Acidi nitrici concentrati partem unam. Immitte in vas vitreum longo collo instructum et adjice Aquæ destillatæ partes quatuor. Mixtis adde, hydrargyri partes quatuor.

"Expone in balneo arenæ caloris gradui th. Fahr. 120—140 (40—48° th. R.) tamdiu donec vesiculæ in superficie liquoris nullæ amplius appareant, id est, donec actio acidi in metallum cesset.—Dein ignem auge ad 200–210° th Fahr. (75–78° th. R.) et sic continua per tres aut quatuor horas; tunc bulliat massa per mediam horam. Dein liquorem bullientem in vas vitreum effunde, in quo prævie 50 partes aquæ destillatæ positæ fuerint, et probe agitando misce.—

"Si durante coctione aut digestione crystalli formantur, illico aqua fervida affundatur et pro re nata etiam pauxillum acidi nitrici.—Ad finem operationis hydrargyri pars in fundo maneat; vel si totum solutum fuerit, addatur portio hydrargyri.

"Solutio mox dicta filtretur eique instilletur gradatim ammoniaca, donec nil amplius pulveris nigri præcipitetur. Pulvis niger, aqua destillata frigida probe edulcoratus, per papyrum album filtretur et perfecte siccetur. Massa siccata papyro griseo duplici involvatur et pondere gradatim aucto probe comprimatur ita ut aqua omnis exprimatur.—Dein pulvis extensus super papyrum gradu 70° th. Fahr. (17° th. R.) siccetur sed non majori.

"USUS: Pro parandis variis compositis.

"DOSIS: Grana duo—sex—octo de die."

The

The single article under *manganese* is the black oxyd, and its alleged use (besides assisting in the oxygenated muriatic acid) is to be externally applied as an ointment for cutaneous eruptions, but its use is doubtful.

Under *Stannum*, the pulvis stanni is properly designated as the *Oxydum Stanni Griseum*.

Under *Stibium*, Mr. Justamond's arsenical caustic of antimony and arsenic is given with approbation.

The emetic tartar, the author prepares with the white oxyd or powder of algaroth, but as this triple salt of antimony, potash, and tartareous acid is found often to vary in its composition, the simple tartrite of antimony is recommended in preference. It is prepared merely by dissolving the white antimonial oxyde in tartareous acid, and evaporating the solution to dryness.

To the metallic salts succeed the *Sulphurets*, the only one of which worthy of remark, is the sulphuret of ammonia, made by passing sulphurated hydrogen gas through liquid caustic ammonia. The gas may be extricated either by sulphuret of iron and muriatic acid, or simply by heating the liquid sulphuret of pot-ash.

A few preparations under the title of *Sapones* follow. The intimate union of soap with the more powerful gum-resins, has, with reason, been thought to improve their efficacy, and render them more soluble in the stomach They are also convenient to be exhibited in the form of pills. These are very intimately mixed by being together dissolved in alcohol, as the author prescribes, and the solution evaporated to a pillular consistence.

The class of *Inflammabilia* includes charcoal, sulphur, phosphorus, resins, oils, and animal fats. The purification of camphor by solution in alcohol and precipitation, is a needless refinement. The following is an imitation of the Indian volatile oil of camphor, so much extolled in rheumatic and gouty cases as an external application. " Mix half a pound of camphor with two pounds of dry pipe clay, and distill in a glass retort with a very slow fire. An acid liquor passes into the receiver, together with a fine volatile oil, and a thick butyraceous oil concreted in the neck of the retort. The volatile oil is to be separated and kept for use.

Among the æthers is given Klaproth's *Acetated Ethereal Tincture of Iron*. It is nine ounces of liquid acetite of iron, mixed with three ounces of acetic ethereal spirit, the preparation of which is also given. A medicine of similar virtues is the nervine tincture, made with muriated iron and sulphuric ether. The whole section on ethers is valuable.

The article *gaza* (gasses) is much too scanty to give any real assistance to the preparer (oxygen excepted).

Ths composition of a few of the most celebrated of the natural or factitious mineral waters is added.

The second part of this Pharmacopœia, is a large selection of formulæ for the use of the practitioner, arranged according to the form of exhibition. Of these, many are selected from various sources sources of acknowledged authority, both in this country and on the continent; the Pharmacopœias of Copenhagen, London, Edinburgh, Stockholm, and Berlin, have furnished others, and a few appear to originate from the author's own experience. In general they are simple, elegant, and well put together, and the number is sufficient to afford a much greater variety than any practitioner is in the habit of employing. The use and the dose of each formula are added in a few words. The following will serve as an example.

"SOLUTIO CAMPHORÆ AQUOSA. ℞. Aquæ acido carbonico saturatæ libras duas. Camphoræ drachmas duas. Misce.

"N. B. Camphora per se in aqua non solubilis, aqua gaze acido carbonico saturata perfecte solvitur, et sic medicamentum præstat egregium in cardialgia spasmodica, singultu, vomitu rebellibus.

"*Vel:* ℞. Camphoræ drachmam unam. Alcoholis quantum opus. Ut fiat solutio, cui adde Aquæ destillatæ fervidæ, libras duas. Probe simul agitatum liquorem cola.

"*Vel:* ℞. Camphoræ, Gummi-resinæ myrrhæ, ana drachmam unam. Simul probe tere, et paulatim adde Aquæ destillatæ fervidæ libram unam. Frigefactum liquorem cola.

"Usus præcipue pro ægris qui camphoram formâ pulveris vel alcohole solutam fere non possunt; Blechropyræ; spasmi; cardialgia; stranguria.

"Dosis; Uncia una—duæ, sæpius de die."

The names of diseases are chiefly those of Sauvages, of which the English reader, who is more familiar with those of Cullen, should be aware. The names given to the materia medica are, as before mentioned, those of Linnæus and of the French chemical nomenclature: and, in some instances, the passion for including a definition in a name has led the author to a ludicrous degree of periphrasis, witness his *Substantia unguinosa (vulgo moschus dicta); Nidus mollis zoophyti, (spongia officinalis); Involucrum reticularis nuclei nucis myristicae moschatae, &c.*

A new remedy proposed for intermittent fever by M. Seguin, (a tanner of Paris, well known by his valuable chemical inquiries into the principles of his art) is here added, with expressions of great confidence in its efficacy. The remedy is the simplest possible, being only a clarified solution of glue, mixed with sugar into a palatable jelly. The following is the formula.

"GELATINA AD DIALEIPYRAS. ℞. Glutinis animalis indurati vulgo venalis libram unam. Aquæ libras sex. Coque ut fiat solutio, cui adjice Albuminis ovi quantum satis Dein massæ probe despumatæ et clarificatæ adde Sacchari albi libram unam. Coque leni igne ad libras tres. Gelatinam sic paratam effunde ut concrescat.

"Usus: Dialeipyræ variæ. Hoc remedium simplex et mite ad dialeipyras varias radicaliter curandas efficacissimum nuperrime invenit Armand Seguin.

"Dosis *infantibus:* Drachmæ duæ-quatuor. *Mediae aetatis et delicatulis:* Drachmæ quatuor-duodecim. *Adultis:* Drachmæ

duodecimduodecim-quadraginta. Prima dosis exhibenda est mox incipiente paroxysmo, præmissis, si opus, præmittendis; et tempore apyrexiæ ter die, continuando per aliquot dies post-quam febris cessavit. Plerumque intra paucos dies febris radicaliter tollitur."

The author announces a Pharmacopœia Chirurgicalis as ready for publication, to which the utility of the present work makes us look with expectation of deriving real advantage.

